# Low glycemic index diet, exercise and vitamin D to reduce breast cancer recurrence (DEDiCa): design of a clinical trial

**DOI:** 10.1186/s12885-017-3064-4

**Published:** 2017-01-23

**Authors:** Livia S.A. Augustin, Massimo Libra, Anna Crispo, Maria Grimaldi, Michele De Laurentiis, Massimo Rinaldo, Massimiliano D’Aiuto, Francesca Catalano, Giuseppe Banna, Francesco Ferrau’, Rosalba Rossello, Diego Serraino, Ettore Bidoli, Samuele Massarut, Guglielmo Thomas, Davide Gatti, Ernesta Cavalcanti, Monica Pinto, Gabriele Riccardi, Edward Vidgen, Cyril W.C. Kendall, David J.A. Jenkins, Gennaro Ciliberto, Maurizio Montella

**Affiliations:** 1 National Cancer Institute Istituto Nazionale Tumori “Fondazione Giovanni Pascale”, IRCCS, Naples, Italy; 2 Clinical Nutrition and Risk Factor Modification Centre, St. Michael’s Hospital, Toronto, Canada; 3 Department of Biomedical and Biotechnological Sciences Oncologic, Clinical and General Pathology Section, University of Catania, Catania, Italy; 4 Cannizzaro Hospital, Catania, Italy; 5 San Vincenzo Hospital, Taormina, Italy; 6 National Cancer Institute CRO, Aviano, Italy; 7 Seconda Universita’ di Napoli, Naples, Italy; 8 Clinica Mediterranea SpA, Naples, Italy; 9 Rheumatology Unit, University of Verona, Verona, Italy; 10 Department of Clinical Medicine and Surgery, Federico II University, Naples, Italy; 11 Department of Nutritional Sciences, Faculty of Medicine, University of Toronto, Toronto, Canada; 12 College of Pharmacy and Nutrition, University of Saskatchewan, Saskatoon, Canada; 13 Division of Endocrinology and Metabolism, St. Michael’s Hospital, Toronto, Canada; 14 Li Ka Shing Knowledge Institute, St. Michael’s Hospital, Toronto, Canada; 15 National Cancer Institute IRCCS Istituto Nazionale Tumori “Regina Elena”, Rome, Italy

**Keywords:** Dietary glycemic index, Mediterranean diet, Exercise, Vitamin D, Breast cancer recurrence, Disease-free survival, Cardiovascular disease, Diabetes, Risk factors

## Abstract

**Background:**

Mechanisms influencing breast cancer (BC) development and recurrence include hyperglycemia, hyperinsulinemia, high insulin-like growth factor-1, high circulating estrogen, inflammation and impaired cellular differentiation/apoptosis. A lifestyle program that targets all the above mechanisms may be warranted. Low glycemic index (GI) foods produce lower post-prandial glucose and insulin responses and have been associated with lower BC risk. Moderate physical activity post-diagnosis reduces BC recurrence and mortality, partly explained by reduced insulin and estrogen levels. Vitamin D increases cell differentiation/apoptosis and high serum vitamin D levels improve BC survival. Yet no trial has evaluated the combined effect of a low GI diet, moderate physical activity and vitamin D supplementation on BC recurrence in the context of a Mediterranean lifestyle setting.

**Methods:**

Women (30-74 yr) who had undergone surgery for primary histologically confirmed BC (stages I-III) within the previous 12 months, in cancer centres in Italy, will be randomized to follow, for a maximum of 33 months, either a high intensity treatment (HIT) composed of low GI diet + exercise + vitamin D (60 ng/mL serum concentration) or a lower intensity treatment (LITE) with general advice to follow a healthy diet and exercise pattern + vitamin D to avoid insufficiency. Both interventions are on a background of a Mediterranean diet. Considering a 20% recurrence rate within 3 years for BC cases and a predicted rate of 10% in the HIT group, with power of 80% and two-sided alpha of 0.05, the subject number required will be 506 (*n* = 253 in each arm). Clinic visits will be scheduled every 3 months. Dietary and exercise counselling and vitamin D supplements will be given at each clinic visit when blood samples, anthropometric measures and 7-day food records will be collected.

**Discussion:**

DEDiCa study aims to reduce BC recurrence in women with BC using a lifestyle approach with additional vitamin D and to investigate possible cardio-metabolic benefits as well as epigenetic modifications according to lifestyle changes. Given the supporting evidence and safety of the components of our intervention we believe it is feasible and urgent to test it in cancer patients.

**Trial registration:**

May 11, 2016; NCT02786875.

**EudraCT Number:**

2015-005147-14

## Background

Breast cancer (BC) is the most common cancer in women and the 5-year survival rate in Europe is 82% suggesting a considerable residual risk [1]. BC is associated also with other chronic conditions including type two diabetes which may increase the risk of BC recurrence [2–4] and many BC patients are at increased risk for cardiovascular disease (CVD) [5, 6]. Mechanisms of BC appear to be linked to sex hormones, impairment in glucose metabolism, hyperglycemia, hyperinsulinemia, insulin-like growth factors (IGF), inflammation, oxidative stress and impaired cell apoptosis [7, 8]. Current advice to BC survivors suggests adopting cancer prevention strategies [8, 9], however there is no consensus on the effectiveness of lifestyle programs in women with BC [10–12] mostly due to lack of sufficient evidence [13]. The rationale for the study is to target several mechanisms of cancer suppression or proliferation, with a healthy diet and exercise program, to avoid low vitamin D levels in order to obtain maximal efficacy of the lifestyle program, and concomitantly to offer maximal CVD and diabetes protection. Dietary carbohydrates are the main food components to affect glycemia and insulinemia. The glycemic index (GI) is able to capture the difference between those that increase glycemia the most (high GI foods) and those that increase it the least (low GI foods) [14]. Low GI foods lower the glycemic and insulinemic potential of the diet and have been shown to reduce the risk of several cancers particularly diabetes-related cancers including BC [15–18] and some evidence suggests they also correlate with lower recurrence [19]. Furthermore, low GI diets have been inversely associated with risk of type two diabetes [15, 20, 21] and CVD [22, 23] and favourably modified blood glucose [24–26], blood lipids [27], inflammatory markers [28], oxidative damage [29], body weight [30] and IGF binding proteins [31], all factors relevant to carcinogenesis, diabetes and CVD. Our previous studies in Italy have shown significant risk reduction of 40% with a low GI diet compared to a high GI diet in BC primary prevention [18]. Physical activity is one of the mainstays of primary prevention of cancer and it is also included in guidelines for BC survivors (at least 150 minutes per week) mainly to reduce complications such as lower muscle strength and risk of depression [13]. However, physical activity after BC diagnosis has also been shown to reduce the risk of BC mortality by 40–50% particularly when it is of moderate intensity such as 30 minutes of brisk walking per day [32–35] an effect possibly modulated partly by insulin economy improvements in reduced insulin, insulin-like growth factors and estrogen levels [36].

To maximize lifestyle changes it may be useful to avoid vitamin deficiencies, particularly vitamin D deficiency which has been linked to higher breast cancer risk [37, 38]. Vitamin D alters genes implicated in cellular growth, affecting proliferation, apoptosis, differentiation, angiogenesis, invasion and metastasis [37]. Preliminary studies suggest that normal-high ranges of serum vitamin D levels improve BC survival [39, 40].

Low glycemic index diet, exercise and vitamin D intake, in addition to determining metabolic changes and benefits for cancer patients, may be able to change the tumor microenvironment and lead to epigenetic modifications [41–44]. In this context, microRNAs (miRNAs), small noncoding RNA molecules, may play a fundamental role in modulating gene expression and breast cancer progression [45, 46]. The evaluation of circulating miRNAs is useful to identify the change of specific miRNAs involved in cancer pathways and predict the development of recurrence in breast cancer patients.

A lifestyle program that targets all the above mechanisms may be warranted. To our knowledge no trial has evaluated the combined effect of a lifestyle program with a healthy diet focusing on low GI, additional physical activity and supplemental vitamin D on BC recurrence and complications in the context of a Mediterranean dietary setting.

## Methods/Design

### Aims and objectives

DEDiCa study aims primarily at reducing BC recurrence in women with BC using a lifestyle approach with additional vitamin D. The primary objective of DEDiCa study is to determine the effect of a 33-month program combining advice on diet, exercise and supplemental vitamin D, on reducing BC recurrence rates or increasing disease-free survival (DFS). Secondary objectives are to improve markers of diabetes risk and management for those who already have diabetes, to improve cardiometabolic health and quality of life (QoL) and to investigate whether changes in microRNA correlate with changes in lifestyle.

### Study design

**Fig. 1:**
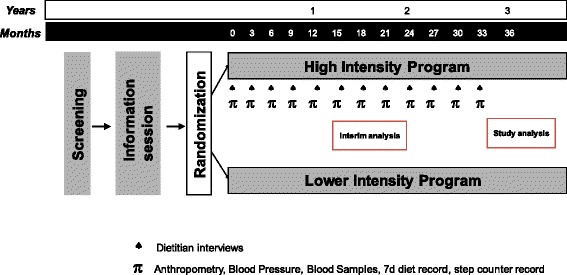
Schematic representation of DEDiCa study protocol

This is a randomized clinical trial targeting women with BC stages I-III within 12 months from BC surgery (see Tables 1 and 2 for details). The study involves at least five cancer centers in Italy (Table 3) and it started on Oct 10^th^, 2016. Consenting participants are randomized to either a high intensity program (HIT) or a lower intensity program (LITE, positive control). The endpoints will be assessed at baseline and every three months until the end of the study (Fig. 1).

**Table 1:** Inclusion and exclusion criteria

Inclusion criteria	Exclusion criteria
1. Women with primary diagnosis of histologically confirmed breast cancer (T1 with Ki67 ≥ 30%, T2, T3 without metastasis) within 12 months from diagnosis. 2. Age ≥ 30 and < 75 years. 3. Patients who are able to comprehend and are willing to sign the consent form and are able to adhere to the protocol including scheduled clinic visits and assigned treatment.	1. Patients who do not possess the inclusion criteria for this study. 2. Patients with sarcoidosis or other granulomatous diseases or with hypercalcemia (Ca > 11 mg/dL). 3. Patients with any previous or current concomitant malignant cancer. 4. Pregnant or lactating women. 5. Patients with AIDS diagnosis 6. Patients with severe renal insufficiency 7. Patients with kidney stones (nephrocalcinosis or nephrolithiasis) 8. Patients participating in other lifestyle clinical trials

**Table 2:** Details of inclusion criteria n. 1

Stage	Primary Tumor	Lymph nodes	Metastasis	Ki-67
I	T1b, T1c	N0	M0	≥30%
IIA	T1a, T1b, T1c T2	N1 N0	M0 M0	any
IIB	T2 T3	N1 N0	M0 M0	any
IIIA	T1a, T1b, T1c T2 T3	N2 N2 N1, N2	M0 M0 M0	any
IIIC	T1a, T1b, T1c T2 T3	N3 N3 N3	M0 M0 M0	any

**Table 3:** List of recruiting centres in Italy

Recruiting centres
• Coordinating Centre: National Cancer Institute Fondazione G. Pascale (Napoli); ○ Via Mariano Semmola – 80131 Napoli ○ Tel: 081/5903395 ○ Email: epidemiologia@istitutotumori.na.it ○ www.istitutotumori.na.it/ • Clinica Mediterranea, Senology Unit; Via Orazio, 2–80122 Napoli; www.clinicamediterranea.it/ • Cannizzaro Hospital, Senology Unit; Via Messina, 829–95126 Catania; www.aocannizzaro.it/ • San Vincenzo Hospital of Taormina, Oncology Unit; Via Sirina, 98039 Taormina (Messina); www.oncologiataormina.it/ • National Cancer Institute CRO Aviano; Via Franco Gallini, 2–33081 Aviano (Pordenone); www.cro.it

To improve compliance participants on both sides will be followed equally with blood tests and clinic visits and will be equally offered general advice on a healthy Mediterranean diet and physical activity, step counters and vitamin D supplements to avoid insufficiency. Participants in the HIT group will additionally receive dietary counselling on how to reduce the GI of their diet, packages of pasta, more in-depth advice on exercise and more vitamin D supplements compared to the LITE group.

This study was approved by the ethic board of the Italian Medicine Agency (AIFA), and of each recruiting hospital: National Cancer Institute “Fondazione Giovanni Pascale” in Naples, Azienda Ospedaliera per l’Emergenza Ospedale Cannizzaro in Catania, Azienda Ospedaliera Universitaria Policlinico “G. Martino” in Messina for San Vincenzo Hospital of Taormina, Comitato Etico Campania Centro ASL NA1 Centro for Clinica Mediterranea in Naples. The study has been registered with ClinicalTrials.gov (registration date and number: May 11, 2016; NCT02786875).

### Participants

Women who had undergone surgery for primary histologically confirmed BC, stages I-III (see Tables 1 and 2 for details), within the previous 12 months in cancer centres in Italy, who are between the ages 30–74 years and have no contraindications to participate in this study will be eligible to participate. Eligibility is confirmed by central reviewing of medical records and pathology reports. The inclusion and exclusion criteria are summarized in Table 1. To participate in the study each potential participant is required to read and sign the study information/consent form at baseline.

### Recruitment and randomization

Eligible participants are recruited in five oncologic centres in Italy (four in Southern Italy and one in Northern Italy). Details of the recruiting centres can be found in Table 3. Eligible participants are contacted either by phone or during one of their follow-up visits and offered to hear more about the study during an information session (either group session or a one-to-one session to accommodate all needs). Eligible participants are explained the study details and are given an informed consent document to take home which they bring back at the baseline visit. At the baseline visit, after obtaining written consent the participant is sent for a blood test and other measurements, is randomized to one of the two arms of the study, receives the advice on the program according to her randomization arm and is given the next appointment slip. Participants are contacted by phone monthly for the first three months and once between visits afterwards. Study visits are scheduled every 3 months until end of study (33 months).

Randomization is done electronically in real time for all recruiting centres and is stratified by stage (I/IIA vs. IIB/IIIA) and age (<50 yrs or ≥50 yrs) at diagnosis and is based on a permuted block design with block size of 4. Stratification by stage and age is done because we expect these variables to affect the outcomes. To prevent any possible study bias the randomization sequence will be done off-site by a third party statistician (Contract Research Organization, Naples, Italy) who will not have contacts with the study participants. The PI is blinded to the randomization of the study participants but not the staff involved in the clinic visits.

### Interventions

Eligible and consenting subjects are randomized to either one of the two treatment programs (higher or lower intensity):

- **Higher intensity (HIT) arm (test)**: low GI diet + exercise + vitamin D.
  1. All carbohydrate foods advised will be low GI choices (GI < 70, on the bread scale), e.g. legumes, pasta al dente, barley, low GI rice, low GI bread, oat, apples, oranges, berries, avocado and nuts.
  2. Brisk walk of at least 30 min per day (or approximately 5000 steps) more than the habitual physical activity.
  3. Vitamin D supplement (cholecalciferol) up to 4000 IU/day to reach the upper end of normal blood levels of 25(OH)D (60 ng/ml).
- Lower intensity (LITE) arm (positive control): general recommendations for a healthy diet and physical activity. Vitamin D (cholecalciferol) will be given only if hypovitaminosis D is detected to bring blood levels up to normal ranges of 30 ng/mL.

Both groups will be counselled to follow a healthy Mediterranean diet (≥5 servings vegetable/fruit per day, ≤1 serving red meat/cold cuts per week, <7% saturated fat).

Treatment evaluations are conducted every three months at each clinic visit and include all three components of the treatment (diet, exercise, vitamin D). Seven-day food records is collected from each patient which is filled a week before the clinic visit. The information in the food record is reviewed by the nutritionist staff with the patient and subsequently inserted in a diet analysis program (WinFood Medimatica, Version 3.9.0). Daily means of GI, energy intake, macro- and micro-nutrients and food groups will be obtained from WinFood and periodically evaluated to ensure adherence to the dietary advice. The physical activity component is measured with a step counter with 7-day memory (OMRON Walking Style IV) and with a questionnaire. Participants bring the step counter back at each clinic visit and the 7-day values are recorded by the research staff. Vitamin D is evaluated by blood analysis of 25(OH)D every three months, the dose reviewed at each clinic visit and changed if necessary to reach the group target (Table 4). QoL is measured with questionnaires specifically made for cancer patients (EQ-5D-3 L, EORTC QLQ-C30 e EORTC QLQ-BC23) [47–49].

**Table 4:** Vitamin D algorithm

Blood levels (ng/ml)	Oral dose (IU)	Treatment duration
Group A (target: 60 ng/ml):		
< 10	75 000 at study visit +4000/day	3 months then re-evaluate
> 10-20	50 000 at study visit +4000/day	3 months then re-evaluate
> 20-30	25 000 at study visit +4000/day	3 months then re-evaluate
> 30	4000/day	3 months then re-evaluate
60-80	change to 1000 IU/day	3 months then re-evaluate
Group B (target: 30 ng/ml):		
≤ 10	100 000 at study visit	Re-evaluate after 3 months
≤ 20	75 000 at study visit	Re-evaluate after 3 months
> 20-25	50 000 at study visit	Re-evaluate after 3 months
> 25-29	25 000 at study visit	Re-evaluate after 3 months
≥ 30	0	Re-evaluate after 3 months

### Outcome measures

Figure 1 depicts the timing and frequency of all study measures. Blood analyses, blood pressure, anthropometric measurements and 7-day food records are taken at baseline and every 3 months afterwards until end of study (up to 33 months). Complete blood analyses are done at baseline, 1 year and end of study while blood analysis pertinent to the treatment are done every three months (Table 5). The primary outcome is the percentage of patients alive at end of study without BC recurrence (disease in the same or opposite breast or any metastasis). The primary outcome is assessed at each collaborating centre by the collaborating oncologist and confirmed by hospital pathological results which are communicated to the coordinating centre. Secondary end points include glycemic control including blood glucose and glycated hemoglobin (HbA1c), cardiometabolic variables including body weight, waist circumference, body mass index (BMI), blood pressure, C-reactive protein (CRP) and blood lipids, hormonal measures including insulin, insulin-like growth factor-1 (IGF-1), estradiol, testosterone and sex hormone binding globulin (SHBG), and epigenetic markers (microRNA). Program adherence and any difficulty noticed, medications and medication changes, as well as any unusual or adverse events, including illness or stressful issues, that occurred since the last clinic visit are recorded in detail.

**Table 5:** Blood analyses performed during the study

Parameters	Baseline, 12 mo and study end	Every 3 months
25(OH)D	x	x
Calcium	x	x
Glucose	x	x
HbA1c	x	x
Insulin	x	
Triglycerides	x	
Total Cholesterol	x	
LDL-C	x	
HDL-C	x	
AST/ALT	x	
CRP	x	
Estradiol	x	
Testosterone	x	
SHBG	x	
IGF-1	x	
microRNA	x	
For future analyses	x	x

*AST/ALT*: aspartate transaminase/alanine transaminase, *CRP*: C-reactive protein, *HbA1c*: hemoglobin A1c, *HDL-C*: high density lipoprotein cholesterol, *IGF-1*: insulin-like growth factor-1, *LDL-C*: low density lipoprotein cholesterol, *SHBG*: sex hormone binding globulin, *25(OH)D*: 25-hydroxyvitamin D.

Blood samples and all information regarding the patient are sent to the coordinating centre where blood samples are centrally analyzed and information entered electronically and statistically analyzed for the interim and end of study reports.

### Biochemical analyses

Serum vitamin D and IGF-1 are analyzed using DiaSorin kits on Liaison XL analyzer (DiaSorin) by chemiluminescent method (CLIA). The HbA1c value is analyzed using whole blood collected in EDTA Vacutainer tubes (Vacutainer; Becton, Dickinson and Co) by a turbidimetric inhibition latex immunoassay (TINA QUANT Roche Diagnostics) on Cobas C6000 analyzer (Roche). Serum glucose, total cholesterol, triglycerides, high-density lipoprotein cholesterol (HDL-C), low-density lipoprotein cholesterol (LDL-C), are measured using reagents and analyzer (Cobas C6000) by Roche Diagnostics according to the manufacturer’s instructions. Serum insulin, estradiol, testosterone and SHBG are performed on the same analyzer by electro-chemiluminescent method (ECLIA). Nephelometric quantification of CRP is performed on BNP ProSpec nephelometer (Siemens Healthcare Diagnostics) according to the manufacturer’s instructions. Serum samples are obtained by blood collected in Vacutainer tubes without anticoagulant (Becton, Dickinson and Co) and analyzed within 24 hours. All analytes are measured in the coordinating hospital routine analytical laboratory undergoing quality control procedures.

### MicroRNA Analysis

Previous studies have demonstrated that microRNAs (miRNAs) are frequently dysregulated in human cancers, including BC [50, 51] and may be modified by the glycemic load of the diet [41]. Computational models are important for the understanding of biological systems [52]. The following are the procedures to identify and analyze miRNA. These analyses will be conducted at the Laboratory of the Biomedical Sciences Department at the University of Catania (Italy).

#### Identification of plasma miRNA in BC

Plasma samples are randomly selected for the analysis by the Human Serum & Plasma miScript miRNA PCR Array (Qiagen) that profiles the expression of 84 miRNAs. In this phase, miRNA expression patterns are analyzed in BC patients according to the lifestyle and dietary intake.

#### Circulating microRNA analysis

miRNAs can be easily purified from a number of patient body fluids. Several studies shown that miRNAs were present in serum and plasma and easily detectable by a sample of peripheral blood. Circulating miRNAs may be important players in the formation of the tumor microenvironment and metastatic evolution by promoting epithelial to mesenchymal transition (EMT) of tumor cells. RNA and miRNAs fraction are extracted from 200 μL of plasma are isolated by miRNeasy Serum/Plasma Kit (Qiagen, Hilden, Germany) according to the manufacturer’s recommendations Extracted miRNAs are reverse transcribed into cDNA and analyzed by miScript SYBR Green PCR Kit (Qiagen).

#### Validation of circulating miRNAs in BC tissues

Data obtained in the previous two steps above are validated in tumor tissue samples to confirm the origin source of the circulating miRNA previously identified.

#### miRNAs and epithelial mesenchymal transition (EMT)

miRNAs, a class of small non-coding RNA molecules that post-transcriptionally regulate gene expression, are attractive candidates for regulating stem cell self-renewal, cell fate decisions and cell plasticity. For this reason, miRNAs analysis is carried out to shed light on the post-transcriptional control of EMT and stemness.

#### Analysis of EMT genes

Publicly available gene expression datasets are analyzed to identify pathways involved in EMT. For this reason a comparative analysis of expression of master regulators of EMT genes (Beta-Catenin, OPN, Twist1/2, Snail1/2, Zeb1/2, N-cad, Vim, E-cad and NGAL) will be performed. GSEA (Gene Set Enrichment Analysis http://broad.mit.edu/gsea) will assess whether the potential pathways involved in EMT, identified in the previous analysis, will be confirmed using a different computational approach. The key genes, representing the highly connected hubs in the identified networks, are assessed by qRT-PCR and IHC on selected archival tumor series to determine their diagnostic and prognostic value according to clinical information, lifestyle and dietary intake.

#### MiRNAs and their role in modulating EMT

Validated miRNAs are analyzed by in silico approaches (miRanda, miRbase, TargetScan) to evaluate if EMT genes previously analyzed are included among their putative target genes. miRNAs here identified and their putative target genes are analyzed in BC tumor tissue, positive for canonical mesenchymal markers, such as Vimentin and N-cadherin. Evaluation of EMT markers are carried out by IHC followed by laser micro-dissection. Further functional experiments are performed to validate in silico considerations.

#### Risk recurrence in BC by computational modeling

The development of a computational model based on agent based modelling, differential equations or Petri nets could lead to a validated tool able to predict the efficacy of the dietary regimen. The model is fit with data coming from the diet regiment components, diet effects, life style, and from plasma miRNAs analysis. miRNAs expression allows the model to be tuned to find evidences that may be relevant to predict the time and the probability of recurrence risk.

### Sample size

Considering a 20% recurrence rate within 3 years in most collaborating centres for BC cases specified in Table 2, and a predicted rate of 10% in the high intensity arm, with power of 80% and two-sided alpha of 0.05, the number of subjects are 506 (*n* = 253 in each arm).

### Statistical analyses

All randomized patients will be analysed considering the “intention-to-treat” principle (ITT analysis). Results will be expressed as percentages or means ± SEM or 95% confidence intervals (CI).

#### Primary analyses

The primary analyses will assess the between-treatment difference in BC recurrence measured as disease-free survival (DFS), calculated as the percentage of patients alive without recurrence of disease at study end (up to 33 months from randomization). BC recurrence is defined as the relapse of the disease or metastasis either in the same breast (including new positive lymph nodes), or the opposite breast or in distant organs. The duration of DFS in patients lost at follow-up will be censured at the date of the last day the patient was considered free of disease. Between-treatment differences in DFS will be analyzed by *log-rank* test. Kaplan-Meier curves will be provided to estimate median DFS and 95% CI. To address the impact of potential imbalance in prognostic factors we will repeat the primary analysis using the *log-rank* test stratified by stage, age, family history of BC, time since diagnosis, molecular subtype, medication use, smoking, baseline waist circumference and dietary variables (baseline GI, dietary fiber, saturated fat, vegetables/fruit, meat, sweets/desserts). Missing data for covariates will be handled by using the missing indicator method.

#### Sensitivity analyses

To assess the robustness of our ITT primary analysis with possible missing data we will repeat the primary analysis using completers data only as well as per-protocol data only, using multiple imputation method to generate missing data in the stratification step. To assess the impact of participant-level factors on the primary outcome we will examine DFS separately in those who, at study end, have reached normo-glycemia (<110 mg/dl) and normal levels of HbA1c <6.0% versus those who have higher levels, in those who reached circulating 25(OH)D ≥60 ng/ml versus those who reached ≤30 ng/ml, in those who have lower versus those who have higher insulin levels (median will be used as cut-offs), in those who have good overall compliance on the three treatment components that is in the highest quantile of: low GI + number of daily steps + levels of 25(OH)D, versus those who have lower compliance. For these analyses study end is defined as the mean of the last three visits.

#### Secondary analyses

The mean and standard error for each of the following variables will be determined for each study group. The change from baseline to end of study will be compared between groups using repeated measures ANOVA and using nonparametric tests if necessary, for the following variables: body weight, waist circumference, BMI, blood pressure, serum levels of 25(OH)D, blood glucose, HbA1c, insulin, IGF-1, lipids, CRP, estradiol, testosterone, SHBG, specific microRNAs, dietary glycemic index, number of steps per day and quality of life. We will also test treatment differences in medication use and medication side effects. Chi-square test will be used to compare categorical variables and Student t-test or Wilcoxon test for continuous variables. The association between each variable and prognosis will be analyzed using Cox proportional hazard model and logistic regression. Finally, we will assess whether these secondary analyses are different at year 1 compared to end of study. All statistical analyses will be conducted with SPSS statistical software version 23.0 (SPSS Inc., Chicago IL, USA).

### Potential toxicity and adverse events

Toxicity due to diet, moderate exercise or vitamin D supplementation are not expected. The diet is an enhanced Mediterranean diet with one arm consuming more low GI foods and physical activity involves moderate exercise of 30 min of daily brisk walk and both respond to lifestyle principles suggested by cancer guidelines such as the American Cancer Society [8] and the World Cancer Research Fund [9]. Oral vitamin D (cholecalciferol) supplementation will be given at safe dosages (up to 4000 IU/day) to reach safe blood levels (60 ng/ml for the test arm and 30 ng/ml for the control arm) [53, 54]. In Italy the normal range of 25(OH)D is between 30-100 ng/ml. Excess vitamin D levels (>100 ng/ml) could induce excess calcium absorption from the intestine and potentially result in hypercalcemia (>11 mg/dl). Both serum 25(OH)D and calcium levels are monitored throughout the study at each clinic visit and any signs or symptom of hypervitaminosis D (hypercalcemia, excess thirst, etc.) will be recorded and immediately communicated to the participant’s physician and supplementation stopped. All adverse events occurring after signing the consent form will be recorded in a specific Adverse Event Form.

### Ethical considerations

The study will determine which group will benefit the most however both treatments are expected to gain some health benefits since both treatments are based on a healthy diet and lifestyle and sufficient vitamin D levels. An interim analysis will be conducted to evaluate whether there are excessive disadvantages for one group over the other. Should this happen the study would be terminated and both groups allowed to follow the most beneficial treatment program. Participants health complications will be dealt by the research team of doctors and by contacting patients’ physicians. Should any complication put the participant at risk by continuing the study the participant will be invited to withdraw from the study. The study participants will also have the right to withdraw from the study anytime and for any personal reason without jeopardizing their health care in the study institution or any other institution.

## Discussion

The purpose of this study is to reduce BC recurrence and hence increase disease-free survival through a lifestyle program that includes a low glycemic index diet, physical activity and vitamin D supplementation in women with BC living in a Mediterranean country. It is expected that the higher intensity program of low GI diet, exercise and vitamin D will reduce BC recurrence by 50% compared to the lower intensity program.

Dietary clinical trials to reduce BC recurrence have been conducted previously. In the USA two large studies were conducted, the Women’s Intervention Nutrition trial (WIN) and the Women’s Healthy Eating and Living trial (WHEL). The WIN study which focused on a low-fat diet found a 24% reduction in BC recurrence after 5 years compared to a control of minimal dietary counselling [10], whereas the WHEL study focusing on a combination of low-fat and high-fruit and -vegetable diet did not reduce BC recurrence after a 7-year intervention compared to a lower intensity fruit and vegetables advice [11]. This null result may be partly explained by methodology aspects as most women had early stage BC and one of the inclusion criteria was the diagnosis of BC within 4 years. It is possible that such a diet may be protective if consumed earlier, possibly within 1 year of diagnosis as in the WIN study [10]. However, in the WHEL study, women who at baseline consumed more than 3 servings of vegetables per day showed a 30% reduction in BC recurrence and an even greater reduction (up to 52%) in tamoxifen users [55]. Furthermore, women who at baseline consumed at least 5 portions of vegetables/fruit per day and additionally walked for at least 30 min a day had a 44% higher chance of survival, independently of obesity, suggesting that obesity did not impact on lowering survival if people adhered to a healthy lifestyle pattern [56]. Overall these large clinical trials suggest the importance of intervening early after diagnosis and that plant-based diets and half hour daily walk may be protective from future recurrence. The low fat advice may however be appropriate only in countries with high intakes of saturated fat. In Mediterranean countries where diets are characterized by high intakes of olive oil, reducing fat intakes may not be beneficial. In the PREDIMED intervention trial, women without BC but following the intensive Mediterranean dietary advice with high olive oil intakes showed up to 68% lower risk of BC compared with those on lower olive oil intake [57]. The traditional Mediterranean diet, rich in plant food and olive oil, has been associated with protection from BC risk [58, 59] CVD events [60], type two diabetes risk [61] and complications [62, 63] and favour weight loss [64]. However, adherence to a traditional Mediterranean diet has halved from the 60’s to 2003 in Mediterranean countries including Italy [65], hence there may be health benefits in improving the current Mediterranean style dietary pattern. The Italian diet is very rich in carbohydrates, particularly bread and other fast absorbing carbohydrates (high GI foods) which have been associated with higher risk of BC as well as other chronic conditions [15, 16, 18, 66]. Since factors influencing the metabolism of glucose may play a relevant role in the development of chronic diseases including BC [15], it is possible that lowering the GI of the Mediterranean diet of Italian women with BC through guided dietary advice, a lower recurrence may be achieved. Chronic elevation of insulin concentrations may be one of the mechanisms explaining the positive association between dietary GI and cancer risk [67]. High GI diets increase blood glucose and insulin levels more than low GI diets and hence may be involved in increasing IGF-1 bioavailability [31]. Insulin is an anabolic hormone able to increase IGF-1 synthesis and activity and IGF-1 in turn may promote cancer development by inhibiting apoptosis, stimulating cell proliferation and sex-steroid synthesis [68, 69]. Another mechanism for high GI-related increased cancer risk may be through hyperglycemia-induced oxidative stress [70, 71] which has been implicated in free radical-dependent DNA damage, known as a contributor to carcinogenesis [72, 73]. Within normal ranges of glycemia higher levels of normal have been directly associated with BC risk in previously healthy women [74] and with higher recurrence rates in women with BC [75]. Another potential mechanism may be through lowering the availability of circulating glucose following low GI diets and exercise. Cancer cells are avid consumers of glucose due to their altered metabolism characterized by insufficient oxidative phosphorylation and compensatory glucose fermentation (the Warburg effect) [76]. This results in lactic acid production and higher protons which acidify the external cellular microenvironment reaching a pH of 6.5-6.9 [77]. A lower pH may provide a competing survival and metastatic advantage for cancer cells (e.g. greater spreading capacity) over normal cells unable to survive below a pH of 7.2 [77, 78] and may induce drug resistance of weak base-anticancer drugs (e.g. doxorubicin) [78].

Physical activity after BC diagnosis has also been shown to reduce the risk of BC mortality by 40-50% particularly when it is of moderate intensity such as 30 minutes of brisk walking per day [32–35]. This effect may be modulated partly by reduced insulin, insulin-like growth factors and estrogen levels [36, 79, 80] which are associated with BC recurrence and death [81, 82]. Furthermore, physical activity can improve insulin sensitivity, reduce blood triglycerides, blood pressure and body fat [83–85]. The lifestyle changes proposed in this study (lower dietary GI, enhanced Mediterranean diet, physical activity) may also induce weight loss which in turn may improve insulin sensitivity, IGF profile and reduce aromatase activity in adipose tissue with consequent reduction in estrogen levels [86], particularly relevant in postmenopausal women [87]. Although weight loss is not a goal of the DEDiCa study, participants will be allowed to lose weight in situations of overwheight or obesity should they wish to. The secondary statistical analyses will take into consideration this aspect.

Lifestyle changes may be more efficacious in a setting of vitamin D sufficiency. Vitamin D alters genes implicated in cellular growth, through upregulation of E-cadherin thereby stimulating cell differentiation and apoptosis [39]. Higher serum vitamin D levels (>30 ng/ml) in BC patients have been associated with 50% less fatality rates compared to lower levels (<20 ng/ml) [88]. The vitamin D dose–response relationship for BC appears inverse and linear up to 60 ng/ml [39]. These levels could achieve a 25% lower BC incidence and could be reached by supplementing 2000–4000 IU/day [53, 89]. The upper dose recommended by the National Academy of Sciences is 4000 IU/day [54]. The Italian guidelines for the prevention and treatment of hypovitaminosis D (SIOMMMS) indicate that in Italy this condition is present in 50% of young adults and at higher rates in older individuals [53]. The updated SIOMMMS guidelines for vitamin D deficiency (25-OH-D < 10 ng/ml) suggest up to 600,000 as a cumulative dose [90]. In Italy the normal range for circulating 25-OH-D is set between 30 ng/ml and 100 ng/ml while toxicity levels are considered above 150 ng/ml [53]. Vitamin D can also protect against bone loss and risk of fractures as a consequence of osteoporosis typically seen after postmenopause or after estrogen deprivation therapy used in BC treatment. Hence vitamin D supplementation may also reduce adverse skeletal effects [53]. Furthermore, higher vitamin D levels have also been associated with reduced risk of developing diabetes [91, 92]. Continuous improvements in survival rates will have an impact on comorbidities and quality of life of BC survivors. Co-morbid conditions have been found at higher prevalence in cancer survivors than in age-matched controls [93] and in the subgroup of cancer patients presenting with two comorbidities, the most frequent combination of diseases appeared to be CVD in men and diabetes in women [94]. There are downstream effects of cancer therapy (e.g. radiation and chemotherapy) causing heart, respiratory, kidney and memory problems but also of hormone suppressing therapy in BC patients [95]. Compounding on this problem, cancer survivors fail to receive the same level of care for their comorbid condition compared to the general population [96] and this is especially true for type two diabetes [97]. Hence it may become even more relevant to implement cost-effective lifestyle risk reduction strategies. Two international cancer organizations have published lifestyle guidelines for cancer survivors, The American Cancer Society (ACS) [8] and the World Cancer Research Fund (WCRF) [9], however there is no agreement on a specific lifestyle program that could reduce BC recurrence and complications including recommendations on vitamin D supplementation.

DEDiCa study includes treatment components such as low GI, traditional Mediterranean foods, high dietary fiber and physical activity which have been shown to reduce risk factors for type two diabetes and CVD including HbA1c, blood glucose and lipids, and inflammatory factors [15, 20, 22, 23, 25–28, 30, 60, 98–100].

This study will contribute to understanding the efficacy of lifestyle changes in a Mediterranean population of BC survivors with respect to several novel aspects: testing a lifestyle modification (diet and exercise) within normal vitamin D levels on disease-free survival; investigate lifestyle changes in relation to BC staging, molecular subtypes, menopausal status, body weight, CVD risk factors and events, diabetes control, new diabetes cases, quality of life, response to medication. It will also allow to understand breast carcinogenesis and the role of microRNA in BC and whether they are modulated by dietary and other lifestyle aspects. It will allow to investigate best time of treatment adherence since women will be enrolled within 12 months of surgery, some will have just started their cancer therapy and some will have ended.

Given the supporting evidence of important health effects and safety of the components of DEDiCa intervention we believe it is feasible and urgent to test this program in BC patients.

## Abbreviations

25(OH)D: 25-hydroxyvitamin D
AST/ALT: Aspartate transaminase/alanine transaminase
BC: Breast cancer
CRP: C-reactive protein
CVD: Cardiovascular disease
GI: Glycemic index
HbA1c: Hemoglobin A1c
HDL-C: High density lipoprotein cholesterol
HIT: High intensity treatment
IGF-1: Insulin-like growth factor-1
LDL-C: Low density lipoprotein cholesterol
LITE: Low intensity treatment
RNA: Ribonucleic acid
SHBG: Sex hormone binding globulin
